# Characterization of Simultaneous Evolution of Size and Composition Distributions Using Generalized Aggregation Population Balance Equation

**DOI:** 10.3390/pharmaceutics12121152

**Published:** 2020-11-27

**Authors:** Mehakpreet Singh, Ashish Kumar, Saeed Shirazian, Vivek Ranade, Gavin Walker

**Affiliations:** 1 Department of Chemical Sciences, Bernal Institute, University of Limerick, V94 T9PX Limerick, Ireland; Saeed.Shirazian@ul.ie (S.S.); Vivek.Ranade@ul.ie (V.R.); Gavin.Walker@ul.ie (G.W.); 2 Pharmaceutical Engineering, Faculty of Pharmaceutical Sciences, Ghent University, 9000 Gent, Belgium; ashish.kumar@ugent.be; 3 Laboratory of Computational Modeling of Drugs, South Ural State University, 76 Lenin Prospekt, 454080 Chelyabinsk, Russia

**Keywords:** aggregation, finite volume scheme, cell average technique, mixing of components, integral moments

## Abstract

The application of multi-dimensional population balance equations (PBEs) for the simulation of granulation processes is recommended due to the multi-component system. Irrespective of the application area, numerical scheme selection for solving multi-dimensional PBEs is driven by the accuracy in (size) number density prediction alone. However, mixing the components, i.e., the particles (excipients and API) and the binding liquid, plays a crucial role in predicting the granule compositional distribution during the pharmaceutical granulation. A numerical scheme should, therefore, be able to predict this accurately. Here, we compare the cell average technique (CAT) and finite volume scheme (FVS) in terms of their accuracy and applicability in predicting the mixing state. To quantify the degree of mixing in the system, the sum-square $\chi^{2}$ parameter is studied to observe the deviation in the amount binder from its average. It has been illustrated that the accurate prediction of integral moments computed by the FVS leads to an inaccurate prediction of the $\chi^{2}$ parameter for a bicomponent population balance equation. Moreover, the cell average technique (CAT) predicts the moments with moderate accuracy; however, it computes the mixing of components $\chi^{2}$ parameter with higher precision than the finite volume scheme. The numerical testing is performed for some benchmarking kernels corresponding to which the analytical solutions are available in the literature. It will be also shown that both numerical methods equally well predict the average size of the particles formed in the system; however, the finite volume scheme takes less time to compute these results.

## 1. Introduction

Population balance equations (PBEs) describe the behavior of particle properties’ changes due to phenomena such as nucleation, growth, aggregation and breakage [1]. Many applications of PBEs can be found in the area of chemical engineering [2], depolymerization [3,4], waste water treatment [5], bubble columns [6], physics [7] and pharmaceutical sciences [8,9,10,11,12], where these mechanisms have a significant impact on the particle properties in the system. In the aggregation process, two or more smaller particles merge at a specific rate to build larger size particles. Aggregation-driven wet granulation processes such as twin-screw granulation [11,13], high shear granulation [14] and fluidized-bed granulation [15] are extensively used in pharmaceutical manufacturing when characterized using PBEs, mostly assuming that only one property of the particles is changing. However, the aggregation mechanism is caused by the mixing-driven presence of liquid binder in the system. Therefore, for such processes, the ability to mechanistically characterize aggregation requires consideration of the degree of mixing between particles and the binder phase. Iveson [16] has shown that the univariate PBEs are inadequate to capture the actual particle behavior in granulation and therefore suggested the application of multivariate (higher dimensional) PBEs. Such PBEs can be used for the simultaneous prediction of several components, the compositional distribution and hence the accurate prediction of the mixing state in the system. Quantification of the degree of mixing in binary component aggregation has been discussed by Matsoukas et al. [17]. The study concluded that the scaling of the variance indicates that the mixing of components is not characterized by a time scale but by a size scale. This emphasizes the need for accurate prediction of particle size distribution evolution, which depends on the selection of a suitable numerical scheme to solve the PBEs and aggregation.

In this study, we focus on the relevance of different numerical schemes for multivariate PBE solution in terms of their capability to capture the degree of mixing during aggregation. In the aggregation mechanism, the total number of particles reduces with time, but the total mass of the system remains constant. A pure bivariate aggregation population balance equation is an integro-partial differential equation which can be written as follows [18].

(1) $$\begin{array}{c} \frac{\partial n(u,t)}{\partial t}=\underset{\mathrm{birth}\;\mathrm{term}}{\underbrace{\frac{1}{2}\int_{0}^{u}a\left(u-u^{\prime },u^{\prime },t\right)n\left(u-u^{\prime },t\right)n\left(u^{\prime },t\right)du^{\prime }}}-\underset{\mathrm{death}\;\mathrm{term}}{\underbrace{\int_{0}^{\infty }a\left(u,u^{\prime },t\right)n\left(u,t\right)n\left(u^{\prime },t\right)du^{\prime }}}, \end{array}$$

subject to the initial condition

$$\begin{array}{c} n\left(u,0\right)=n_{0}\left(u\right),\;\;\;\;\;u\in \left[0,\infty \right]. \end{array}$$

Here, n(u,t) is the number distribution functions having size u≥0 at time *t* (s) and u (μm) is a vector given in terms of the summation of properties like the mass or volume of the components. The bold notations are used to denote vector quantities, i.e., $u=[u_{1},u_{2},\cdots ,u_{p}]$, where $u_{p}$ expresses the property of the particle in pth direction. Moreover, the birth term in Equation (1) represents the formation of new particles of properties u due to the aggregation of smaller particles of properties $u-u^{\prime }$ and $u^{\prime }$. Similarly, the death term describes the loss of particles with properties u due to the collision of particles with properties $u^{\prime }$. The aggregation kernel $a(u,u^{\prime },t)$ describes the rate of merging of two particles of properties u and $u^{\prime }$. It can be noted that the aggregation kernel is non-negative and symmetric in nature with respect to size variable, i.e., $a\left(u,u^{\prime },t\right)=a\left(u^{\prime },u,t\right)$. Moreover, for simplicity, we assume that the aggregation kernels chosen for this study are time-independent, i.e., only size dependency is taken into consideration.

Apart from finding the number distribution function *n*, different properties of the system, namely integral moments corresponding to the number distribution function, are also important for understanding the complete dynamics of the system [19]. For bivariate PBE, the $\left\{i_{1},i_{2},\cdots ,i_{p}\right\}\mathrm{th}$ order moment corresponding to the number distribution function is defined as

(2) $$\begin{array}{c} \mu_{i_{1},i_{2},\cdots ,i_{p}}\left(t\right)=\int_{0}^{\infty }\prod_{r=1}^{p}u_{r}^{i_{r}}n\left(u,t\right)du. \end{array}$$

Here, $\mu_{0,0,\cdots ,0}\left(t\right)$ denotes the total number of particles in the system, which is also known as zeroth-order moment, whereas $\mu_{0,0,\cdots ,1,\cdots ,0}\left(t\right)$ gives the total mass of the *p*th property. Similarly, other moments can also be obtained.

Finding analytical (exact) solutions of the population balance equation (PBE) (1) is difficult due to the presence of a nonlinear integral in the equation. However, still, for some simple structured kernels, a few analytical solutions are listed in [20,21,22,23]. Therefore, in this exercise, we choose numerical approximations to solve bivariate pure aggregation PBE (1). To date, many numerical methods have been proposed by various authors, including fixed pivot techniques [24,25,26], method of projection [27], Euler method [28], finite volume schemes [29,30,31,32,33,34,35,36,37,38,39], quadrature method of moments [40,41,42,43], finite element method [44,45], sectional methods [18,46,47,48,49,50] and Monte Carlo method [19,51,52,53].

Among all numerical schemes, the cell average technique (CAT) is well known for the accurate prediction of the number distribution function and their moments. However, the mathematical formulation for CAT is very complex; hence, it is computationally expensive [54,55]. The finite volume schemes (FVS) are well recognized in the literature as being able to accurately and efficiently obtain these results. The studies conducted in the last decade restricted their comparison only to the number distribution functions and their integral moments [18,35,46,47,49,56]. To examine the accuracy of the prediction of the component mixing for a higher-dimensional PBE, the cell average technique [46] and the finite volume scheme [57] are implemented to approximate a multi-dimensional pure aggregation PBE and compared. The verification of the numerical results is also conducted by comparing the average size of particles predicted in the system using the exact solution.

The paper is organized as follows: to start, a brief introduction of the existing CAT as well as the FVS for solving bivariate pure aggregation PBE on non-uniform meshes is provided in Section 2. In Section 3, the qualitative and quantitative numerical results, particularly various order moments and number density functions computed by both numerical schemes, are analyzed. Finally, Section 4 summarizes the conclusions and discussion of this study.

## 2. Numerical Methods and System Analysis

**Figure 1:**
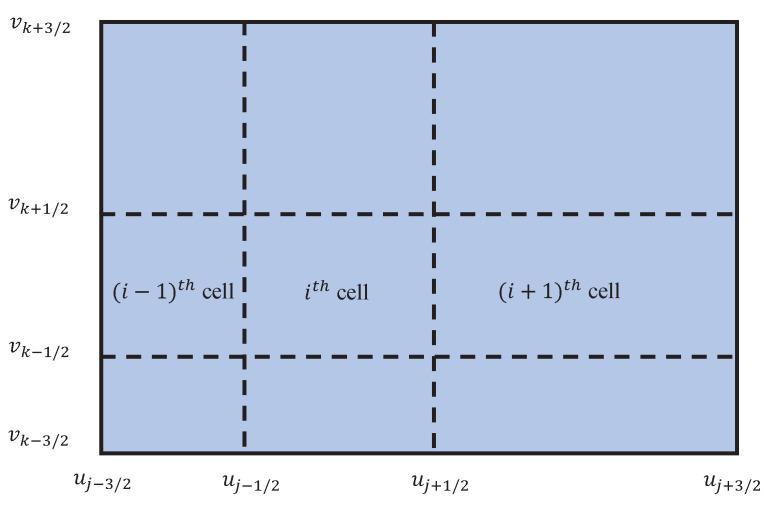
Rectangular domian discretization in 2D space.

In this section, the mathematical formulations of the existing cell average technique [46] and the finite volume scheme [57] for solving a bivariate aggregation PBE (1) on non-uniform grids are outlined (Figure 1). For developing the expressions of both numerical methods, it is assumed that particles within a grid cell are concentrated on its representative (or mean of the cell). Before giving the description of the numerical methods, it is necessary to fix the computational domain. For the numerical approximations, we define the size variables $u^{\prime }$ in PBE (1) ranges from 0 to *∞*; thus, a large sufficient vector such as $u_{max}={\left[u_{1_{max}},u_{2_{max}},\cdots ,u_{p_{max}}\right]}^{T}$ is replaced with *∞* in the second integral of PBE (1). Thus, the original PBE (1) takes the following form:

(3) $$\begin{array}{c} \frac{\partial n(u,t)}{\partial t}=\frac{1}{2}\int_{0}^{u}a\left(u-u^{\prime },u^{\prime },t\right)n\left(u-u^{\prime },t\right)n\left(u^{\prime },t\right)du^{\prime }-\int_{0}^{u_{max}}a\left(u,u^{\prime },t\right)n\left(u,t\right)n\left(u^{\prime },t\right)du^{\prime }, \end{array}$$

with the modified initial condition

$$\begin{array}{c} n\left(0,u\right)=n_{0}\left(u\right),\;\;\;\;\;u\in \left[0,u_{max}\right]. \end{array}$$

The above expression well suited to numerical simulations; however, it does not capture the property of mass conservation. During numerical computations, a sufficiently large $u_{max}$ is chosen to minimize loss of mass from the system. Further, we assume that the whole domain is divided into $I=(I_{1},I_{2},I_{3},\cdots ,I_{p})$, where $I_{r}$ is the number of grids in *r* direction for r={1,2,3,⋯,p}. Now, for any *r*, the mesh points and the step size can be defined by

$$u_{1_{r}-1/2}=0,\;\;\;u_{i_{r}}=\frac{u_{i_{r}-1/2}+u_{i_{r}+1/2}}{2},\;\;\;\Delta u_{i_{r}}=u_{i_{r}+1/2}-u_{i_{r}-1/2}.$$

### 2.1. Cell Average Technique (CAT)

First, the mathematical explanation of the CAT developed by Kumar et al. [46] on the non-uniform grids is presented. Let us suppose that $N_{i}$ defines the number of particles in the *i*th cell, which can be expressed as

(4) $$\begin{array}{c} N_{i}=\int_{u_{i}-1/2}^{u_{i}+1/2}n\left(u,t\right)du, \end{array}$$

where $du=\prod_{r=1}^{p}du_{r}$ and $\int_{u_{i-1/2}}^{u_{i+1/2}}=\prod_{r=1}^{p}\int_{u_{i_{r}-1/2}}^{u_{i_{r}+1/2}}$ for $i=i_{1},i_{2},\cdots ,i_{p}.$ Now, let us express the number distribution function in terms of dirac delta functions, i.e.,

(5) $$\begin{array}{c} n\left(u,t\right)=\sum_{j=1}^{I}N_{j}\delta \left(u-u_{j}\right). \end{array}$$

Substituting the above expression in the original PBE (1), we obtain the following set of ordinary differential equations:

(6) $$\begin{array}{c} \frac{dN_{i}}{dt}=B_{i}-D_{i}\;\mathrm{for}\;i=i_{1},i_{2},\ldots ,i_{p}. \end{array}$$

Here, the discrete forms of birth and death terms are expressed as

(7) $$\begin{array}{c} B_{i}=\sum_{u_{i-1/2}\leq \left(u_{j}+u_{k}\right)<u_{i+1/2}}^{j\leq k}\left(1-\frac{1}{2}\delta_{j,k}\right)a\left(u_{j},u_{k}\right)N_{j}N_{k}, \end{array}$$

and

(8) $$\begin{array}{c} D_{i}=\sum_{j=1}^{I}a\left(u_{i},u_{j}\right)N_{i}N_{j}. \end{array}$$

Here, $j=[j_{1},j_{2},\ldots ,j_{p}]$ and $k=[k_{1},k_{2},\ldots ,k_{p}]$ are *p*-dimensional vectors. The corresponding mass or flux of the particles, which takes birth in the *i*th cell, can be computed as follows:

(9) $$\begin{array}{c} V_{i}=\sum_{u_{i-1/2}\leq \left(u_{j}+u_{k}\right)<u_{i+1/2}}^{j\leq k}\left(1-\frac{1}{2}\delta_{j,k}\right)a\left(u_{j},u_{k}\right)N_{j}N_{k}\left(u_{j}+u_{k}\right). \end{array}$$

Further, once the aggregation events of the particles in the *i*th cell are completed, it is necessary to compute the average properties of all birth events in the cell using the following expression:

(10) $$\begin{array}{c} {\bar{u}}_{i}=\frac{V_{i}}{B_{i}}. \end{array}$$

It is assumed that the particle properties are concentrated on the representative of the cell; however, the possibility of the aggregating particles falling on the representative is low. Therefore, if the birth $B_{i}$ takes place in the *i*th cell and is not represented by the nodes, then the averaging properties, such as $b_{1},b_{2},\cdots ,b_{p}$, are distributed to the neighboring nodes in such a fashion that the integral properties, particularly the zeroth and first order moments for our case, remain conserved. The assigned values $b_{1},b_{2},\cdots ,b_{p}$ can be calculated from the following relations:

(11) $$\sum_{r=1}^{p}b_{i_{r}}=1,\;\;\;\sum_{r=1}^{p}b_{i_{r}}u^{A_{i_{r}}}={\bar{u}}_{i},$$

where $u^{A_{i_{1}}},u^{A_{i_{2}}},\cdots ,u^{A_{i_{p}}}$ are the coordinate vectors of the vertices of the *i*th cell. Hence, the final set of discrete equations can be written as

(12) $$\begin{array}{c} \frac{dN_{i}}{dt}=\sum_{u_{i-1/2}\leq \left(u_{j}+u_{k}\right)<u_{i+1/2}}^{j\leq k}\left(1-\frac{1}{2}\delta_{j,k}\right)a\left(u_{j},u_{k}\right)N_{j}N_{k}-\sum_{j=1}^{I}a\left(u_{i},u_{j}\right)N_{i}N_{j}. \end{array}$$

**Figure 2:**
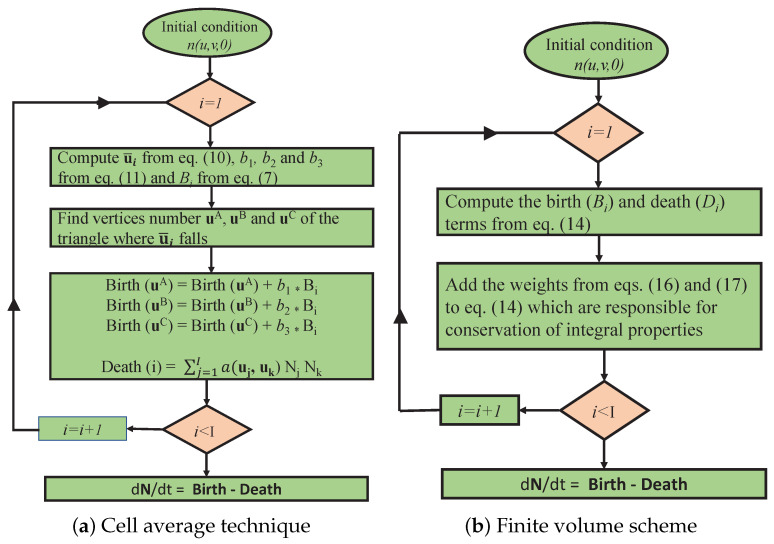
Work flowcharts of the algorithms of the numerical methods.

It can be noted that the cell average technique is computationally very expensive as it is necessary to determine the average properties of the aggregating particles in each cell and then redistribution of particle properties is carried out to the neighboring representative of the cell. The distribution is carried out in such a way that the zeroth and first order moments of the system are conserved. A detailed description of the cell average technique can be found in Kumar et al. [46] and its schematic flowchart is provided in Figure 2a.

### 2.2. Finite Volume Scheme (FVS)

Now, we provide the mathematical formulation of the finite volume scheme [57] for solving a generalized aggregation PBE. The finite volume scheme is based on the idea of preserving the total number of particles and conserving the total mass in the system by just adding two correction factors in the formulation. For developing the mathematical expression of the FVS in a similar domain, it is necessary to define the following set of indices:

(13) $$\begin{array}{c} \equiv^{i}=\left\{\left(j,k\right)\in N\times N:u_{i-1/2}<\left(u_{j}+u_{k}\right)\leq u_{i+1/2}\right\} \end{array}$$

where $u_{i-1/2}$ and $u_{i+1/2}$ denote the boundaries of the *i*th cell, respectively, and $u_{i}$ represents the mean value of the *i*th cell. The representation of the $\equiv^{i}$ defines all those pairs of cell indices *j* and *k* with particle properties $u_{j}$ and $u_{k}$, such that the addition of their particle properties $u_{j}+u_{k}$ will fall in any cell having representative $u_{i}$ after the aggregation of particles.

Similar to the CAT, for the FVS, it is presumed that the point masses are concentrated on the representatives. Therefore, by proceeding in a similar way as in the CAT, the expression for the FVS is obtained, which is given by

(14) $$\begin{array}{c} \frac{dN_{i}}{dt}=\frac{1}{2}\sum_{u_{i-1/2}<\left(u_{j}+u_{k}\right)\leq u_{i+1/2}}^{j\leq k}a\left(u_{j},u_{k}\right)N_{j}N_{k}-\sum_{j=1}^{I}a\left(u_{i},u_{j}\right)N_{i}N_{j}. \end{array}$$

We know that the finite volume schemes are well known for the conservation of the various properties. The above formulation (14) takes into account the preservation of the zeroth order moment but not the conservation of the total mass (first order moment) of the system. Nevertheless, this can be resolved by introducing only two weights in the above equation, leading to the following expression:

(15) $$\begin{array}{c} \frac{dN_{i}}{dt}=\frac{1}{2}\sum_{u_{i-1/2}<\left(u_{j}+u_{k}\right)\leq u_{i+1/2}}^{j\leq k}a\left(u_{j},u_{k}\right)N_{j}N_{k}\varphi_{j,k}^{b}-\sum_{j=1}^{I}a\left(u_{i},u_{j}\right)N_{i}N_{j}\phi_{i,j}^{d}, \end{array}$$

where the correction factors $\varphi_{j,k}$ and $\phi_{i,j}$ are defined as

(16) $$\begin{array}{c} \varphi_{j,k}^{b}=\left\{\begin{array}{cc} \frac{\theta \left(u_{j}\right)+\theta \left(u_{k}\right)}{2\theta \left(u_{l_{j,k}}\right)-\left(\theta \left(u_{j}\right)+\theta \left(u_{k}\right)\right)}, & \left(u_{j}+u_{k}\right)\leq u_{max};\\ 0, & \mathrm{otherwise}. \end{array},\right. \end{array}$$

and

(17) $$\begin{array}{c} \phi_{i,j}^{d}=\left\{\begin{array}{cc} \frac{\theta (u_{l_{i,j}})}{2\theta \left(u_{l_{i,j}}\right)-\left(\theta \left(u_{i}\right)+\theta \left(u_{j}\right)\right)}, & \left(u_{i}+u_{j}\right)\leq u_{max};\\ 0, & \mathrm{otherwise}. \end{array},\right. \end{array}$$

where $\theta (u_{i})$ denotes the sum of the components of the vector $u_{i}$, i.e., $\theta \left(u_{i}\right)=\theta \left(u_{i_{1}}\right)+\theta \left(u_{i_{2}}\right)+\cdots +\theta \left(u_{i_{p}}\right)$ and $l_{i,j}$ is the index of the cell where $\left(u_{i}+u_{j}\right)$ falls. The theoretical proof of the preservation of the total number of particles as well as total mass of the particles in the system along with the CFL conditon can be found in Kaur et al. [57] and its flowchart is depicted in Figure 2b.

### 2.3. Kernel Selection

The efficiency and accuracy of the numerical methods is tested against the analytically tractable kernels corresponding to 2D PBE. In particular, two kernels, namely constant (size-independent) and sum (size-dependent) kernels, are chosen for the comparison.

#### 2.3.1. Size-Independent Kernel

For a size-independent kernel $a(u,v,u^{\prime },v^{\prime })=1$, the analytical solution corresponding to the size-independent kernel was formulated by Gelbard and Seinfeld [22] and is listed in Table 1.

#### 2.3.2. Size-Dependent Kernel

Mathematically, the sum kernel can be expressed as $a\left(u,v,u^{\prime },v^{\prime }\right)=u+v+u^{\prime }+v^{\prime }$ and is heavily dependent on the size of the particles. The analytical solution in this case is formulated by Fernández-Díaz and Gómez-García [21] and is listed in Table 2.

### 2.4. Model Initialization and Post-Processing

For the initial condition n(u,v,0)=16uvexp(−2u−2v), the exact results of number density as well as various order moments for constant and sum kernels are provided in the literature by Gelbard and Seinfeld [22] and Fernández-Díaz and Gómez-García [21], respectively. Before comparing the numerical results, it is important to define the degree of aggregation $I_{agg}$:

(18) $$I_{agg}\left(t\right)=1-\frac{\mu_{00}\left(t\right)}{\mu_{00}\left(t=0\right)}\;,\;t\geq 0\;,$$

which describes the decrease in the number of initial primary particles due to the aggregation process. At time t=0, $I_{agg}=0$, and as it approaches large values, $I_{agg}\to 1$, with all primary particles forming one large particle.

Furthermore, the weighted relative error is also calculated to test the accuracy of the number distribution quantitatively [49]:

(19) $$\Delta_{i,j}\left(t\right)=\frac{\sum_{p=1}^{I_{1}}\sum_{q=1}^{I_{2}}\left|N_{p,q}^{ana}\left(t\right)-N_{p,q}^{num}\left(t\right)\right|\;u_{p}^{i}v_{q}^{j}}{\sum_{p=1}^{I_{1}}\sum_{q=1}^{I_{2}}N_{p,q}^{ana}\left(t\right)u_{p}^{i}v_{q}^{j}}.$$

The superscripts ana and num represent the exact and numerical solutions, respectively. Here, $\Delta_{0,0}$ represents the relative error in the distribution of the number of particles over the whole size domain. The two solutions corresponding to the two numerical schemes may identify the same prediction for the total number of particles, even though the distribution of particle populations may disagree considerably. This is well captured by $\Delta_{0,0}$. Similarly, the $\Delta_{1,0}$ expresses the relative error in the distribution of the total mass of the system. All the simulations and computations for both schemes were carried out using “MATLAB” on a i5 CPU with 2.67 GHz and 16 GB RAM.

### 2.5. Average Size Particles

The average size of particles formed in the system along *u* and *v* components is determined using the following expression:

(20) $$\bar{u}=\frac{\mu_{10}\left(t\right)}{\mu_{00}\left(t\right)},\;\;\;\;\;\;\;\;\;\bar{v}=\frac{\mu_{01}\left(t\right)}{\mu_{00}\left(t\right)}.$$

Moreover, the total average size of particles formed in the system is given by

(21) $$\bar{u}=\frac{\mu_{10}\left(t\right)+\mu_{01}\left(t\right)}{\mu_{00}\left(t\right)}.$$

### 2.6. Quantification of Mixing

For identifying the mixing of components in a bivariate PBE, Matsoukas et al. [17] provided a theory to show that the mixing of components is calculated using the χ parameter corresponding to the variance of excess binder. The underlying principle relies on the assumption that if the components are perfectly mixed among all aggregates, the amount of binder in an aggregate of size *v* would be ϕv. If the actual amount of binder in the aggregate is $u_{i}$, the difference m−ϕv defines the excess binder χ.

For kernels independent of both composition and initial conditions, the $\chi^{2}$ parameter should be constant at all times for constant and sum kernels. The expression for a $\chi^{2}$ parameter is given below:

(22) $$\chi^{2}=\eta^{2}\mu_{20}\left(t\right)-2\eta \left(1-\eta \right)\mu_{11}\left(t\right)+{\left(1-\eta \right)}^{2}\mu_{20}\left(t\right),$$

where η=0.5. A detailed description of the theory of mixing of components can be found in Matsoukas et al. [17] and Matsoukas and Marshall Jr [19].

## 3. Results and Discussion

The efficiency and accuracy of the numerical schemes for 2D PBE solutions are tested using exact solutions applying analytically tractable kernels. For qualitative and quantitative testing of numerical schemes, CAT and FVS are compared in terms of various order moments and number density functions approximated by both numerical schemes. The results are presented separately for two selected kernels, namely constant (size-independent) and sum (size-dependent) kernels.

### 3.1. Size-Independent Kernel

#### 3.1.1. Comparison of Moments and Number Density Prediction

**Figure 3:**
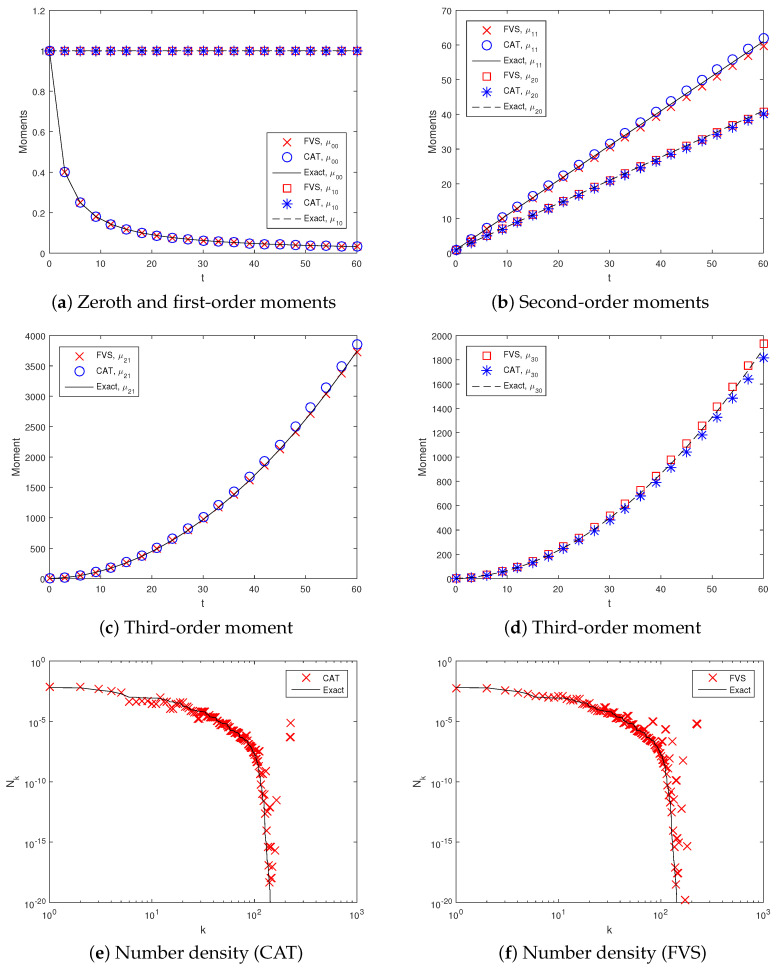
Different order moments and number density functions for constant kernel.

For a constant (or size-independent) kernel, the computational domain is taken from $u_{min},v_{min}=6\times 10^{-5}$ to $u_{max},v_{max}=21$. This domain is partitioned into 20×20 non-uniform cells and time ranging from 0 to 60. Numerical simulations achieved a degree of aggregation $I_{agg}\left(t\right)=0.97$ during this time on a non-uniform grid. Figure 3 shows various order moments predicted by both numerical methods compared to the exact moments. This reveals that the zeroth and first-order (Figure 3a) moments are equally well predicted by both methods and match well with the exact moments. In other words, both numerical approximations ensure the preservation of the total number of particles and the conservation of the total mass of the particles in the system. Additionally, the second-order moments ($\mu_{20}$ & $\mu_{11}$) computed by both numerical methods are in good agreement with the exact moments (Figure 3b). However, the third-order moment, namely $\mu_{30}$, is more accurately computed by the FVS than by the CAT (Figure 3e). Similar behavior is also shown by the other third-order moment ($\mu_{21}$) as FVS shows a more precise result than the CAT, overlapping with the exact result.

In addition to this, using the flat representation concept [58], the number of particles in each cell of the computational domain obtained numerically is compared with the exact results qualitatively. The idea of the flat representation is to sort the cells’ indices from 1 to $I_{1}\times I_{2}$ in ascending order, find the particle population in each cell by the relation $N_{k}=n_{k_{1},k_{2}}\Delta u_{k_{1}}\Delta u_{k_{2}}$ and plot against its pivot index *k*. The plots given in Figure 3e,f show that the CAT more accurately predicts the particle population in each cell, whereas the FVS shows more deviation from the exact solution.

#### 3.1.2. Comparison of Average Particle Size and Mixing State Prediction

**Figure 4:**
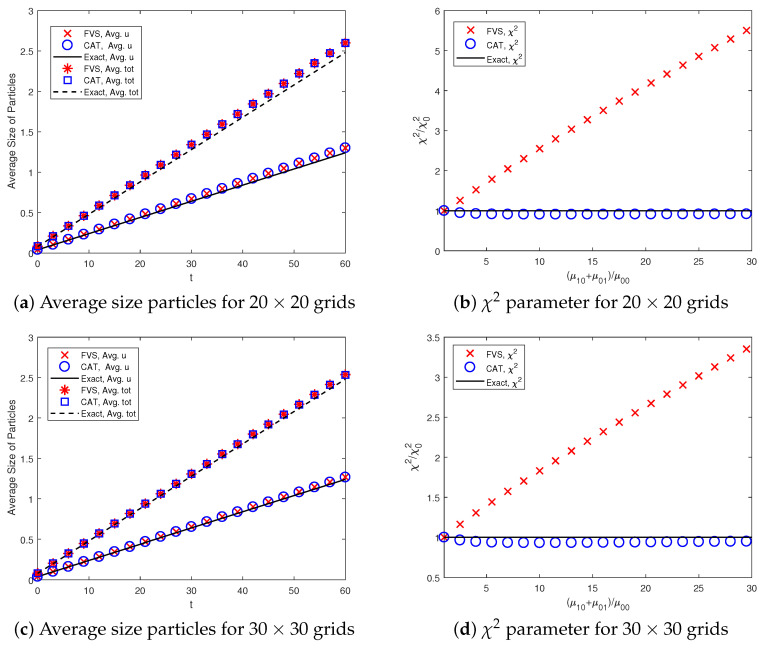
Average size particles formed in the system and $\chi^{2}$ parameter for mixing the components using constant kernel.

The average size of particles formed and mixing of components (variance of excess binder) is calculated using Equations (21) and (22). In order to test the accuracy of prediction by the two methods, the numerical results for the system are compared with the exact results in Figure 4. It can be observed that the results for the average size of particles are equally well computed by both numerical methods (see Figure 4a). This is due to the fact that the accuracy of the average size particles depends on the accuracy of the zeroth and first-order moments and both methods predicted these results with equal precision. Moreover, the variance of excess binder plotted in Figure 4b shows that the CAT is in better agreement with the analytical solution than the finite volume scheme. However, the accuracy of these results can be improved to any level by considering a well-refined grid (as shown in Figure 4c,d).

As a further comparison, the quantitative weighted errors (19) existing in the number distribution functions are also found using the numerical methods (see Table 3). The table below reveals that the weighted relative errors in the distribution of the various moments found using the CAT are larger than the FVS. One can also observe that the weighted errors in various order moments can be enhanced by opting for a more refined grid. In terms of CPU time, the FVS took 30.35 s to obtain all numerical results, whereas the CAT took 35.22 s for a size-independent kernel. Therefore, from the above discussion, it can be concluded that the accurate prediction of the various order normalized moment depends enormously on the behavior of the weighted sectional moments errors but not always on the behavior of the number distribution function.

### 3.2. Size-Dependent Kernel

#### 3.2.1. Comparison of Moments and Number Density Prediction

The same numerical methods that were applied using the constant kernel are repeated using the size-dependent kernel (sum kernel) and the results are compared. The computational domain taken from $u_{min},v_{min}=6\times 10^{-5}$ to $u_{max},v_{max}=30$ is divided into 20×20 non-uniform cells with time varying from 0 to 20. The simulations attained a degree of aggregation of 0.90 at the end time.

**Figure 5:**
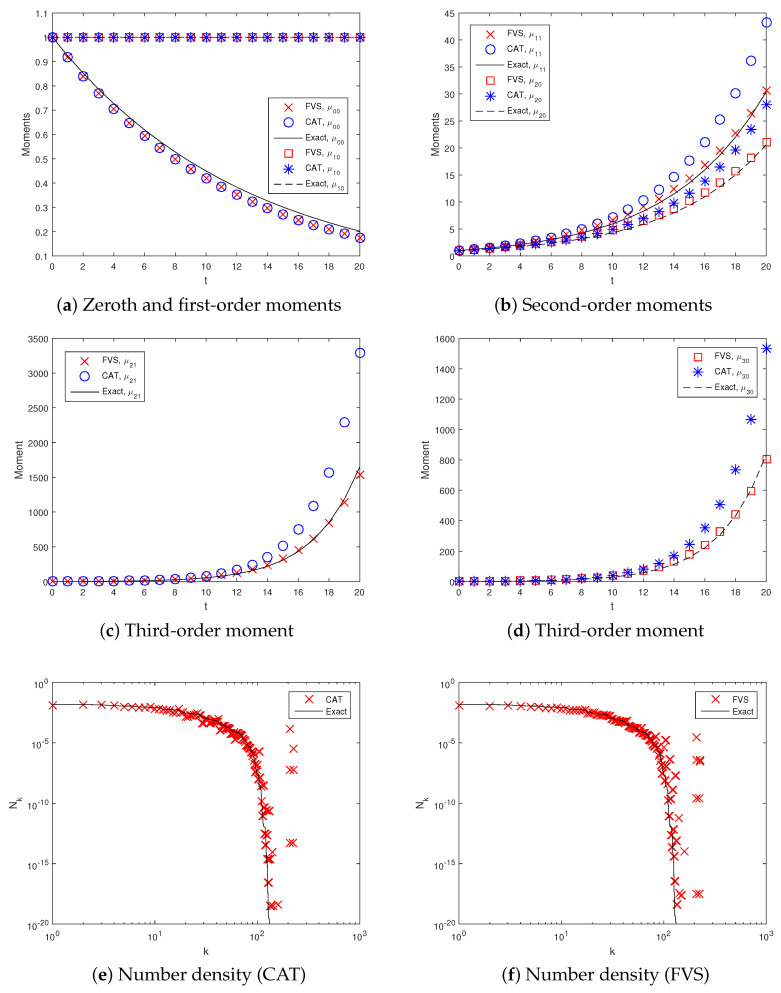
Different order moments and number density functions for sum kernel.

Figure 5 represents the comparison of results obtained using both numerical approximations with the exact results. It can be seen that the zeroth order moments obtained using the FVS and the CAT match well with each other, though both show under-prediction of the exact result (Figure 5a). However, these numerical results can be improved by choosing a more refined grid. The first-order moment predicted by both numerical methods shows good agreement with the exact result, i.e., the total mass of the particles in the system is conserved. Furthermore, Figure 5b reveals that the second-order moments ($\mu_{20}$ & $\mu_{11}$) computed by the FVS show less deviation from the exact moments as compared to the CAT. Similarly, it can be seen from Figure 5c,d that the third-order moments, namely $\mu_{21}$ and $\mu_{30}$, predicted by the FVS, overlap with the exact results, whereas the CAT shows more deviation from the exact moments. The deviations in the moments may be due to the numerical diffusion taking place in the CAT while distributing the averages of the particles to the neighboring nodes, but no such distribution of particles is required in the case of FVS.

Similar to the constant kernel, the particle population in each cell obtained numerically is compared with the exact result using a flat representation for the size-dependent kernel (see Figure 5e,f). These figures reveal that the CAT shows more accurate results than the FVS. In addition to this, the quantitative weighted relative errors in the different order moments computed using both numerical methods are shown in Table 4. The results reveal that the weighted errors existing in the various order moments are larger for the CAT, whereas FVS shows fewer errors. It can also be observed that the errors in the higher-order moments increase substantially more than the lower-order moments for the CAT, whereas the FVS shows stable results. The weighted relative errors in the various moments decrease when a more refined grid is taken into consideration. Consequently, from the above discussion, it can be again concluded that the accurate prediction of the various normalized moments certainly depends on the behavior of the sectional weighted relative errors in the moments.

#### 3.2.2. Comparison of Average Particle Size and Mixing State Prediction

**Figure 6:**
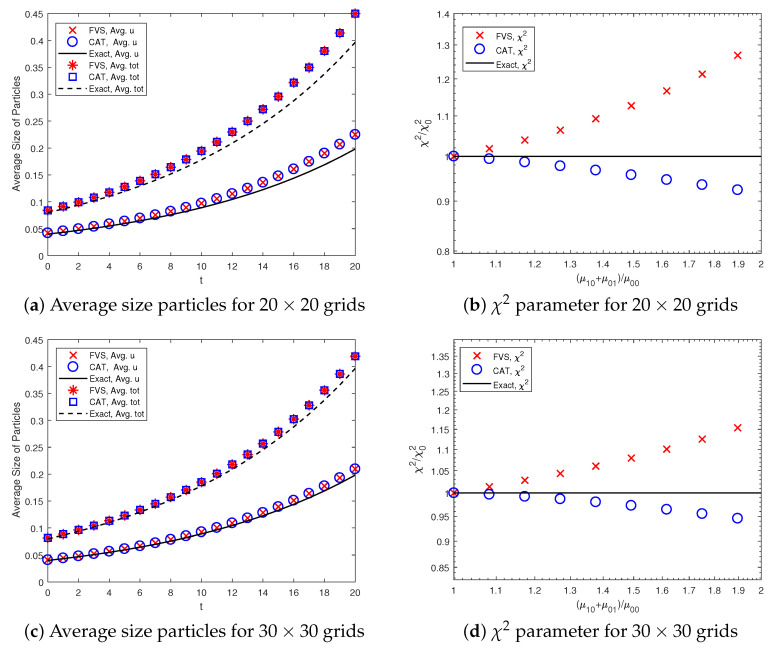
Average size particles formed in the system and $\chi^{2}$ parameter for mixing the components using sum kernel.

The average size particles and variance of the excess binder are compared in Figure 6 for the sum kernel. One can easily observe that the results are similar to the previous case. The average size particles are equally well obtained by both numerical methods, as illustrated in Figure 6a.

However, the excess binder variance is more accurately obtained by the cell average technique, even though the second-order moments are more accurately computed by the finite volume scheme (see Figure 5b). In contrast, the FVS estimates the second-order moments more accurately than the CAT. However, it shows a large deviation from the exact results, as shown in Figure 6b. Using a refined grid, the accuracy of the results can be improved to a large extent, as shown in Figure 6c,d. In a computational sense, the CAT took 20.09 CPU time to approximate the numerical results. However, the FVS required less time to predict these results (approximately 17.91 s).

## 4. Conclusions

This study demonstrates that the accurate prediction of second-order moments does not always predict the variance of the excess binder, which is very important for computing the extent of component mixing in aggregation-driven processes such as pharmaceutical granulation. Two numerical methods, namely the cell average technique and finite volume scheme, have been implemented for solving a multi-dimensional aggregation population balance equation to show these results. Many applications, including granulation and crystallization, merely focus on the accuracy of the various order moments and number distribution function. For this purpose, the finite volume scheme can be used for various practical applications such as granulation, crystallization and bubble columns due to its simpler mathematical formulation and higher accuracy than the cell average technique. However, to compute the extent of the mixing of binder in the system, the accurate prediction of excess binder variance ($\chi^{2}$ parameter) is very important. Even though the cell average technique is less accurate than the finite volume scheme in second- and third-order moment prediction, it is recommended for computing the $\chi^{2}$ parameter as it requires less computational CPU time. The accuracy of the $\chi^{2}$ parameter can be improved to the desired level; however, this is at a computation expense as more grid points would need to be considered in the computational domain.

## Figures and Tables

**Table 1:** Analytical solutions of number density functions for size-independent kernel.

Parameter	Value
$N_{0}$	1
$p_{1},p_{2}$	1
$m_{10},m_{20}$	0.04
n(u,v,t)	$\frac{4N_{0}}{{\left(t+2\right)}^{2}}\frac{{\left(p_{1}+1\right)}^{\left(p_{1}+1\right)}{\left(p_{2}+1\right)}^{\left(p_{2}+1\right)}}{m_{10}m_{20}}\exp\left[\frac{-(p_{1}+1)u}{m_{10}}+\frac{-(p_{2}+1)v}{m_{20}}\right]$
	$\times \sum_{k=0}^{\infty }{\left(\frac{t}{t+2}\right)}^{k}\frac{{\left[{\left(p_{1}+1\right)}^{\left(p_{1}+1\right)}\right]}^{k}{\left[{\left(p_{2}+1\right)}^{\left(p_{2}+1\right)}\right]}^{k}{\left(u/m_{10}\right)}^{\left(k+1\right)\left(p_{1}+1\right)-1}{\left(v/m_{20}\right)}^{\left(k+1\right)\left(p_{2}+1\right)-1}}{\Gamma \left[\left(p_{1}+1\right)\left(k+1\right)\right]\Gamma \left[\left(p_{2}+1\right)\left(k+1\right)\right]}$

**Table 2:** Analytical solutions of number density functions for size-dependent kernel.

Parameter	Value
$N_{0}$	1
$p_{1},p_{2}$	1
$m_{10},m_{20}$	0.04
τ	1−exp(ϕt)
*s*	u+v
$s_{0}$	$m_{10}+m_{20}$
n(u,v,t)	$N_{0}\left(1-\tau \right)\exp(\frac{-s\tau }{s_{0}})\frac{\left(p_{1}+1\right)\left(p_{2}+1\right)}{m_{10}m_{20}}\exp\left[\frac{-(p_{1}+1)u}{m_{10}}+\frac{-(p_{2}+1)v}{m_{20}}\right]$
	$\times \sum_{k=0}^{\infty }\frac{1}{(k+1)!}{\left(\frac{-s\tau }{s_{0}}\right)}^{k}\frac{{\left[\left(p_{1}+1\right)u/m_{10}\right]}^{\left(k+1\right)\left(p_{1}+1\right)-1}{\left[\left(p_{2}+1\right)v/m_{20}\right]}^{\left(k+1\right)\left(p_{2}+1\right)-1}}{\Gamma \left[\left(p_{1}+1\right)\left(k+1\right)\right]\Gamma \left[\left(p_{2}+1\right)\left(k+1\right)\right]}$
ϕ	total mass of the particles in the system

**Table 3:** Quantitative weighted sectional errors for constant kernel.

Moments	CAT	FVS	CAT	FVS
	20×20	20×20	25×25	25×25
$\Delta_{0,0}$	0.27110	0.14838	0.22142	0.10737
$\Delta_{1,0}$	0.29518	0.21496	0.25672	0.14373
$\Delta_{2,0}$	0.36367	0.32288	0.32813	0.25187
$\Delta_{1,1}$	0.35038	0.30358	0.32345	0.24179
$\Delta_{3,0}$	0.51598	0.46139	0.48715	0.42507
$\Delta_{2,1}$	0.53205	0.51217	0.49247	0.45377

**Table 4:** Quantitative weighted sectional errors for sum kernel.

Moments	CAT	FVS	CAT	FVS
	20×20	20×20	25×25	25×25
$\Delta_{0,0}$	0.18180	0.15587	0.11325	0.08356
$\Delta_{1,0}$	0.33015	0.14861	0.30735	0.10185
$\Delta_{2,0}$	0.81804	0.29930	0.65429	0.27372
$\Delta_{1,1}$	0.82524	0.27404	0.65840	0.26817
$\Delta_{3,0}$	2.12448	0.59022	1.43660	0.37667
$\Delta_{2,1}$	2.05260	0.67102	1.40687	0.38896

## Nomenclature

**Symbol**	**Description**
*n*	Particle property (size) distribution
u	Particle property vector (size)
*t*	time
$N_{i}$	Number of particles in the cell *i*
$\mu_{i}$	*i*th order moment
I	Total number of cells
$I_{agg}$	Degree of aggregation
$l_{j,k}$	Index of the cell where $\left(u_{j}+u_{k}\right)$ falls
φ	Weight function
*a*	Aggregation kernel
Δ	Measure of sectional error
θ	Sum function
$\bar{u}$	Average size of particles along *u* axis
$\bar{v}$	Average size of particles along *v* axis
$\chi^{2}$	Mixing of components

## Abbreviations

PBE	Population balance equation
FVS	Finite volume scheme
CAT	Cell average technique
